# Synthesis and evaluation of novel amide amino-β-lactam derivatives as cholesterol absorption inhibitors

**DOI:** 10.1016/j.bmc.2015.03.067

**Published:** 2015-05-15

**Authors:** Tonko Dražić, Vinay Sachdev, Christina Leopold, Jay V. Patankar, Martina Malnar, Silva Hećimović, Sanja Levak-Frank, Ivan Habuš, Dagmar Kratky

**Affiliations:** a Ruđer Bošković Institute, Bijenička cesta 54, Zagreb, Croatia; b Institute of Molecular Biology and Biochemistry, Medical University of Graz, Graz, Austria

**Keywords:** TBDMS, *tert*-butyldimethylsilyl, FCS, fetal calf serum, MTT, 3-(4,5-dimethylthiazol-2-yl)-2,5-diphenyltetrazolium bromide, THAP, 2,4,6 trihydroxyacetophenone, DAC, ammonium citrate dibasic, β-Lactam, Cholesterol absorption inhibitor, Hyperlipidemia, Cardiovascular heart disease

## Abstract

The β-lactam cholesterol absorption inhibitor ezetimibe is so far the only representative of this class of compounds on the market today. The goal of this work was to synthesize new amide ezetimibe analogs from *trans*-3-amino-(3*R*,4*R*)-β-lactam and to test their cytotoxicity and activity as cholesterol absorption inhibitors. We synthesized six new amide ezetimibe analogs. All new compounds exhibited low toxicity in MDCKIIwt, hNPC1L1/MDCKII and HepG2 cell lines and showed significant inhibition of cholesterol uptake in hNPC1L1/MDCKII cells. In addition, we determined the activity of the three compounds to inhibit cholesterol absorption in vivo. Our results demonstrate that these compounds considerably reduce cholesterol concentrations in liver and small intestine of mice. Thus, our newly synthesized amide ezetimibe analogs are cholesterol absorption inhibitors in vitro and in vivo.

## Graphical abstract

**Figure:**
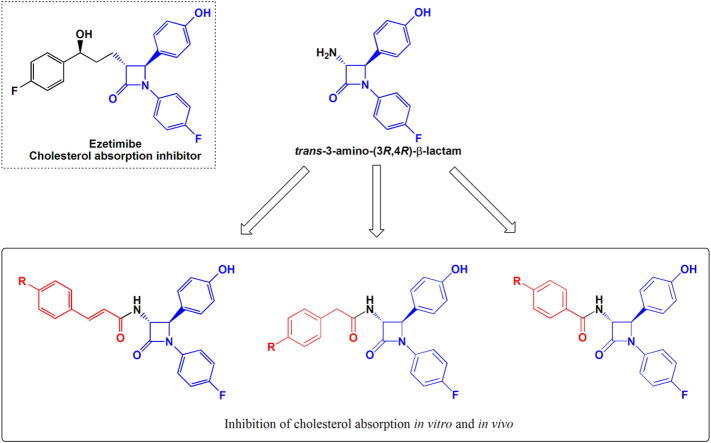

## 1 Introduction

**Figure 1:**
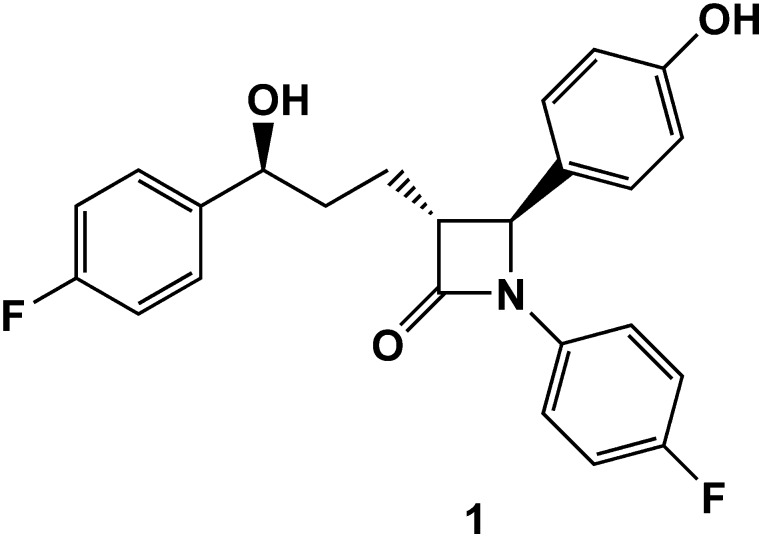
The structure of ezetimibe **1**.

Coronary heart disease (CHD) is a major health concern and a leading cause of death in the world^1^ with high blood cholesterol concentrations being one of the critical risk factors.^2^ There are two major sources of cholesterol in the body: de novo synthesis in liver and absorption of dietary cholesterol in the small intestine.^3^ Lowering of blood cholesterol levels can be achieved by blocking either of the two pathways. De novo synthesis is pharmacologically inhibited with 3-hydroxy-3-methylglutaryl coenzyme A (HMG-CoA) reductase inhibitors (statins),^4^ whereas absorption of dietary cholesterol can be blocked with β-lactam cholesterol absorption inhibitors.^5^ Ezetimibe **1** (Zetia, Ezetrol) (Fig. 1), FDA approved in 2002, is the only representative of this class of compounds on the market today. It can be administered as monotherapy or in combination with statins.^6^ In 2004, Niemann Pick C1-like1 (NPC1L1) protein was identified as a molecular target of ezetimibe.^7^

The following structural characteristics are important for the activity of cholesterol absorption inhibitors: the β-lactam backbone, aryl group at the N-1 position of the β-lactam, aryl group optimally substituted with alkoxy or hydroxy group at *para*-position at the C-4 position of the β-lactam, the side chain with three linking atoms bearing a pendent aryl group at the C-3 position of the β-lactam, and *S* configuration at the C-4 chiral center.^5^ Furthermore, Wang et al. reported that ezetimibe bioisosteres with an amide group at the 3′ position of the C-3 β-lactam side chain display cholesterol absorption inhibitory activity.^8^

**Figure 2:**
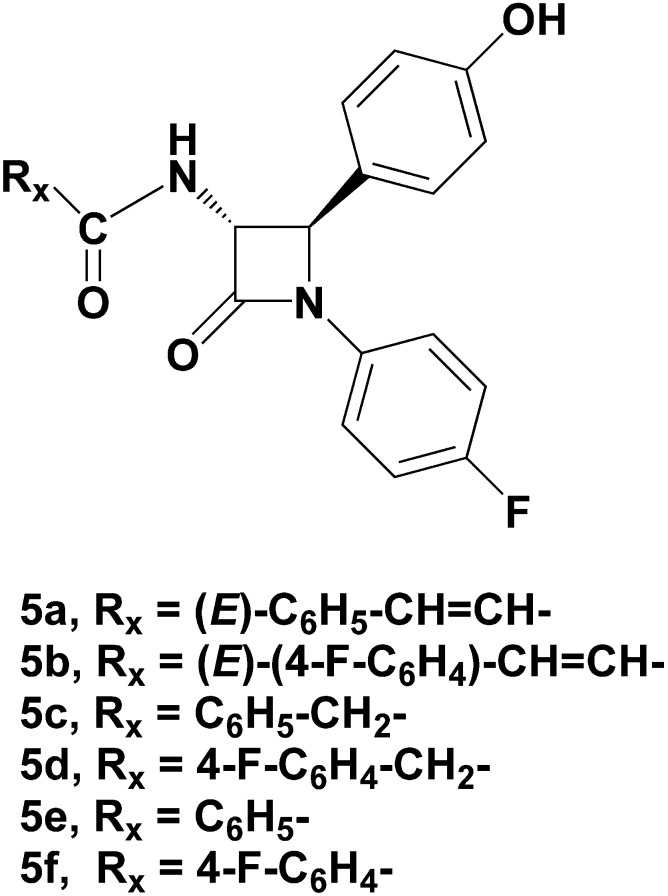
Structure of novel amide ezetimibe analogs **5a**–**f**.

In this report, we investigated if the introduction of an amide group at the C-3 position of the β-lactam ring has an effect on cholesterol absorption inhibitory activity. Furthermore, we wanted to examine if the incorporation of the cinnamoyl group together with β-lactam in a single molecule improves its ability to inhibit cholesterol absorption. Cinnamon, including cinnamaldehyde and cinnamates as its important constituents, is reported to have anti-hyperglycemic and anti-hyperlipidemic properties.^9^, ^10^, ^11^, ^12^ Therefore, we implemented molecular hybridization, a strategy where different bioactive entities are combined into a single molecule,^13^ and combined two constituents with reported effect on cholesterol metabolism. In addition, we investigated whether the length of the side chain containing amide group has a role in the inhibition of cholesterol absorption. For that reason, either phenyl or benzyl substituent was attached to the amide group. In continuation of our research,^14^, ^15^, ^16^, ^17^, ^18^, ^19^ we synthesized six new ezetimibe analogs **5a**–**f** (Fig. 2) from *trans*-3-amino-(3*R*,4*R*)-β-lactam **2** and determined their cytotoxicity, as well as in vitro and in vivo activity.

## 2 Results and discussion

### 2.1 Chemistry

#### 2.1.1 Synthesis of novel amide ezetimibe analogs

**Scheme 1:**
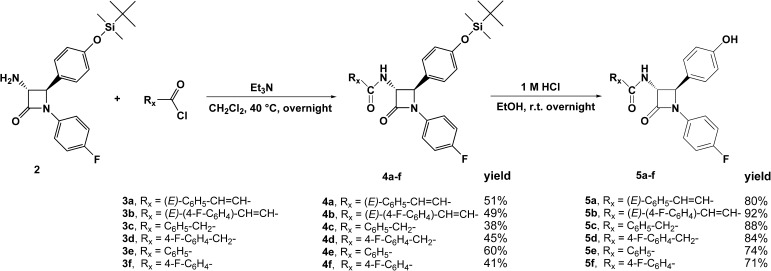
Synthesis of amides **5a**–**f** from *trans*-3-amino-(3*R*,4*R*)-β-lactam **2**.

Enantiomerically pure *trans*-3-amino-(3*R*,4*R*)-β-lactam **2** was synthesized applying the chiral ester enolate-imine condensation.^19^, ^20^ N-acylation of β-lactam **2** was carried out with the corresponding acyl chloride **3a**–**f** in dichloromethane in the presence of Et_3_N at reflux (Scheme 1). OTBDMS protected amides **4a**–**f** were obtained in 40–60% yield. Deprotection was performed with 1 M HCl in ethanol to afford **5a**–**f** in 70–90% yield.

### 2.2 Biological evaluation

#### 2.2.1 Cytotoxicity analysis

Cytotoxicity of ezetimibe **1** and of newly synthesized amides **5a**–**f** was determined using MTT cell proliferation assay in Madine-Darby Canine Kidney II wild-type (MDCKIIwt), MDCKII cells stably expressing hNPC1L1 (hNPC1L1/MDCKII) and HepG2 cell lines (Table 1). hNPC1L1/MDCKII were used as a pharmacologically validated model for investigating NPC1L1-mediated cholesterol uptake.^21^ LC_50_ values were determined and values higher than 100 μM were considered non-toxic. Amides containing cinnamoyl (**5a** and **b**), as well as phenyl group (**5e** and **f**) exhibited low cytotoxicity with LC_50_ values between 55 and 91 μM and were comparable to ezetimibe **1** (Table 1). Amides with benzyl substituent (**5c** and **d**) had LC_50_ values higher than 100 μM. To unambiguously confirm that the tested compounds in combination with micelles do not affect the in vitro cholesterol uptake by a potential cytotoxic effect, we evaluated their combined cytotoxicity in MDCKIIwt and hNPC1L1/MDCKII cells. All tested compounds, when combined with micelles, showed no toxicity (Fig. S1).

**Table 1:** In vitro cytotoxicity of ezetimibe **1** and newly synthesized amide ezetimibe analogs **5a**–**f**^a^

Compound	MDCKII	hNPC1L1/MDCKII	HepG2
**1**	>100	62.29	69.74
**5a**	66.55	65.06	63.27
**5b**	63.95	63.27	57.47
**5c**	>100	>100	>100
**5d**	>100	>100	>100
**5e**	91.23	68.34	67.58
**5f**	78.64	64.69	71.93

a The results are expressed as LC_50_ (μM).

#### 2.2.2 In vitro cholesterol uptake

**Figure 3:**
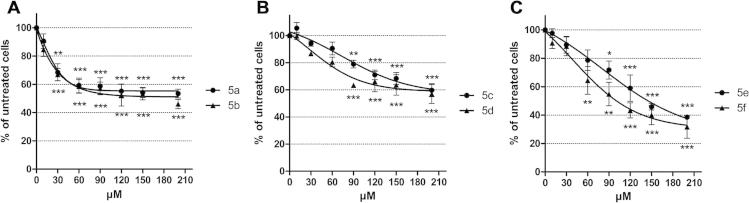
Inhibition of cholesterol uptake in hNPC1L1/MDCKII cells for compounds (A) **5a** (IC_50_ = 20 μM) and **5b** (IC_50_ = 18 μM), (B) **5c** (IC_50_ = 88 μM) and **5d** (IC_50_ = 46 μM), (C) **5e** (IC_50_ = 70 μM) and **5f** (IC_50_ = 54 μM). The results are expressed as percentage of inhibition compared to untreated cells and represent mean ± SEM of three independent experiments. ^∗^*p* <0.05, ^∗∗^*p* <0.01, ^∗∗∗^*p* <0.001 determined by one-way ANOVA followed by Dunnett’s test.

Ezetimibe analogs **5a**–**f** were tested for inhibition of cholesterol uptake in hNPC1L1/MDCKII cells (Fig. 3). The activity of ezetimibe **1** was determined previously (IC_50_ = 24 μM, maximum inhibition of 40% reached at ∼60 μM concentration).^19^ Analogs containing a cinnamoyl group (**5a** and **b**) had lowest IC_50_ values (20 and 18 μM, respectively), with maximum inhibition of ∼45% which was reached at 60 μM concentration (Fig. 3A), and their activity was comparable to ezetimibe **1**. Compounds containing a benzyl substituent (**5c** and **d**) exhibited maximum inhibition of ∼40% with IC_50_ values of 88 and 46 μM, respectively (Fig. 3B). Derivatives with a phenyl group (**5e** and **f**) inhibited cholesterol uptake by 60–65% and had IC_50_ values of 70 and 54 μM, respectively (Fig. 3C). It is important to notice that derivatives containing 4-F-phenyl at the side chain displayed lower IC_50_ values than their phenyl couterparts in all three groups (Fig.3A–C). These results show that amide ezetimibe analogs **5a**–**f** are potent inhibitors of cholesterol uptake in vitro.

#### 2.2.3 In vivo cholesterol absorption

Finally, we tested the in vivo inhibition of cholesterol absorption of ezetimibe **1** and 4-F-phenyl representatives (**5b**,**d**,**f**) from each group of ezetimibe analogs. For in vivo testing, we used the compounds with lower IC_50_ values and higher inhibition of cholesterol uptake as determined in vitro. For that purpose, mice were treated with 20 mg/kg/day of each compound for two days, gavaged with [^3^H]cholesterol and radioactivity was measured in the liver and three equal parts of the small intestine (duodenum, jejunum, ileum). The results are expressed as percent reduction in radioactivity relative to vehicle (Table 2). All tested compounds caused a significant reduction of radioactivity in the liver. Cinnamoyl analog **5b** decreased [^3^H]cholesterol content by 49%, which was comparable to ezetimibe **1** (53%), whereas benzyl (**5d**) and phenyl (**5f**) analogs displayed decreased absorption by 39% and 37%, respectively. In the three parts of the small intestine (duodenum, jejunum, ileum), ezetimibe **1** lowered [^3^H]cholesterol by 60%, 52%, and 44%, respectively. Our newly sythesized compounds lowered [^3^H]cholesterol in all parts of the small intestine in the range of 14% and 42%, not reaching statistical significance in any part of the intestine. Compound **5b** exhibited highest activity with 27%, 35%, and 37% inhibition, respectively. **5d** lowered radioactivity in duodenum and jejunum by 15% and 14%, whereas **5f** showed 42% and 32% inhibition, respectively. Decreased cholesterol absorption in the ileum was comparable between **5d** (24%) and **5f** (28%). These results suggest that all tested compounds alter cholesterol metabolism in vivo with **5b** showing the highest and **5d** resulting in the lowest inhibition of cholesterol absorption.

**Table 2:** In vivo inhibition (%) of cholesterol absorption for compounds **1**, **5b**, **5d**, and **5f**

Compound	Liver	Duodenum	Jejunum	Ileum
**1**	53 ± 2⁎⁎	60 ± 6⁎⁎	52 ± 3⁎	44 ± 11
**5b**	49 ± 5⁎⁎	27 ± 10	35 ± 8	37 ± 15
**5d**	39 ± 11⁎	15 ± 12	14 ± 20	24 ± 13
**5f**	37 ± 8⁎	42 ± 2	32 ± 6	28 ± 7

3–4 mice per group, 20 mg/kg/day for 2 days mean ± SEM.

⁎ *p* <0.05.

⁎⁎ *p* <0.01 determined by one-way ANOVA followed by Dunnett’s test.

## 3 Conclusion

We have successfully synthesized six new amide ezetimibe analogs **5a**–**f** from *trans*-3-amino-(3*R*,4*R*)-β-lactam. All new compounds exhibit low toxicity and significant inhibition of cholesterol uptake in vitro. Furthermore, inhibitory activity of three new amides (**5b**,**d**, and **f**) was tested in vivo. All three compounds considerably lowered cholesterol level in liver and small intestine, with the cinnamoyl analog **5b** being the most active one and the benzyl derivative **5d** showing fewest effects on cholesterol absorption. These results suggest that amide ezetimibe analogs are efficient cholesterol absorption inhibitors and that incorporation of cinnamoyl group as anti-hyperlipidemic moiety contributes to the inhibitory activity.

## 4 Experimental section

### 4.1 General methods

Melting points were determined on a Reichert Thermovar 7905 apparatus and have not been corrected. The IR spectra were recorded on a PerkinElmer Spectrum RX I FT-IR System spectrometer (KBr pellets technique) (PerkinElmer Instruments, Norwalk, CT, USA). The NMR spectra were measured in CDCl_3_ or DMSO-*d*_6_ at RT on a Bruker AV 300 and/or AV 600 spectrometer (Bruker BioSpin GmbH., Rheinstetten, Germany). *δ* is given in ppm relative to tetramethylsilane as an internal reference. Optical rotations were determined on an Optical Activity Automatic Polarimeter AA-10 in 1 dm cell; *c* in g/100 mL (Optical Activity Ltd, Ramsey, England). Silica gel column chromatography was performed using silica gel 70–230 mesh, 60 Å (Sigma–Aldrich, St. Louis, MO, USA or Acros-Organics, New Jersey, USA) at RT. Samples for HR-MS analysis were resuspended in 5 μL of THAP/DAC matrix and 1 μL was spotted onto a MALDI plate. Mass spectra were obtained on a matrix-assisted laser desorption/ionization-time-of-flight (MALDI-TOF/TOF) mass spectrometer (4800 Plus MALDI-TOF/TOF Analyzer, Applied Biosystems, Foster City, CA, USA) equipped with Nd:YAG laser operating at 355 nm with firing rate 200 Hz in the positive ion reflector mode. 1600 shots per spectrum were taken covering a mass range of 100–1000 Da, focus mass of 500 Da, and delay time of 100 ns.

### 4.2 General procedure for the acylation of **2**

The reaction was carried out in dry conditions under argon atmosphere. To a solution of **2** in anhydrous CH_2_Cl_2_ (5 mL), Et_3_N (6.0 equiv) and corresponding acyl-chloride (2.0 equiv) were added. The reaction mixture was refluxed overnight, after which distilled water was added and the corresponding product extracted with CH_2_Cl_2_ (3 × 20 mL). Collected organic layers were dried over Na_2_SO_4_ and solvent evaporated to dryness. The product was purified by silica gel column chromatography (hexane/ethyl acetate 2:1).

#### 4.2.1 (*E*)-1-[(3*R*,4*R*)-1-(4-Fluorophenyl)-4-(4-(*t*-butyldimethylsilyloxy)phenyl)-2-oxo-3-azetidinylamino]-3-phenyl-2-propen-1-one (**4a**)

Obtained from **2** (63 mg, 0.16 mmol) and (*E*)-3-phenylprop-2-enoyl chloride **3a** (55 mg, 0.32 mmol) as white solid (43 mg, yield 51%). mp 189–191 °C; [α]_D_^20^ +106 (*c* 5 mg/mL EtOAc); FT-IR (KBr) cm^−1^: 3448, 3236, 3059, 2929, 2856, 1751, 1618, 1509, 1388, 1357, 1259, 1226, 917; 830; ^1^H NMR (300 MHz, CDCl_3_): 0.19 (s, 6H, Si–(C*H*_3_)_2_), 0.97 (s, 9H, C–(C*H*_3_)_3_), 4.75 (dd, 1H, *J*_1_ = 7.1 Hz, *J*_2_ = 2.2 Hz, C3 β-lactam), 5.05 (d, 1H, *J* = 2.0 Hz, C4 β-lactam), 6.47 (d, 1H, *J* = 15.7 Hz, C*H*=CH–C(=O)–NH), 6.80–6.88 (m, 4H, Ar-*H*), 7.17–7.20 (m, 4H, Ar-*H*), 7.30–7.32 (m, 4H, Ar-*H*), 7.39–7.42 (m, 2H, Ar-*H*, CH=CH–C(=O)–N*H*), 7.58 (d, 1H, *J* = 15.7 Hz, CH=C*H*–C(=O)–NH); ^13^C NMR (75 MHz, CDCl_3_): −4.3 (Si–(*C*H_3_)_2_), 18.3 (*C*–(CH_3_)_3_), 25.7 (C–(*C*H_3_)_3_), 63.3 (C3 β-lactam), 66.4 (C4 β-lactam), 115.9 (d, *J* = 22.9 Hz, 4-F-*C*_6_H_4_), 119.1 (d, *J* = 8.1 Hz, 4-F-*C*_6_H_4_), 119.2 (*C*H=CH–C(=O)–NH), 120.8 (4-OTBDMS-*C*_6_H_4_), 127.6 (4-OTBDMS-*C*_6_H_4_), 128.1 (*C*_6_H_5_), 128.5 (4-OTBDMS-*C*_6_H_4_), 128.9 (*C*_6_H_5_), 130.1 (*C*_6_H_5_), 133.5 (d, *J* = 2.8 Hz, 4-F-*C*_6_H_4_), 134.6 (C_6_H_5_), 142.7 (CH=*C*H–C(=O)–NH), 156.3 (4-OTBDMS-*C*_6_H_4_), 159.4 (d, *J* = 243.9 Hz, 4-F-*C*_6_H_4_), 165.0 (*C*=O, β-lactam), 166.5 (*C*=O); HRMS for C_30_H_33_FN_2_O_3_Si (*M*_r_ = 516.67852): calcd. *m*/*z* [M+Na^+^] 539.2136, found 539.2148.

#### 4.2.2 (*E*)-1-[(3*R*,4*R*)-1-(4-Fluorophenyl)-4-(4-(*t*-butyldimethylsilyloxy)phenyl)-2-oxo-3-azetidinylamino]-3-(4-fluorophenyl)-2-propen-1-one (**4b**)

Obtained from **2** (62 mg, 0.16 mmol) and (*E*)-3-(-4-fluorophenyl)prop-2-enoyl chloride **3b** (59 mg, 0.32 mmol) as white solid (43 mg, yield 49%). mp 182–184 °C; [α]_D_^20^ +106 (*c* 5 mg/mL EtOAc); FT-IR (KBr) cm^−1^: 3448, 3230, 3039, 2956, 2931, 2858, 1749, 1654, 1611, 1510, 1391, 1358, 1267, 1231, 1158, 914, 830; ^1^H NMR (300 MHz, CDCl_3_): 0.19 (s, 6H, Si–(C*H*_3_)_2_), 0.97 (s, 9H, C–(C*H*_3_)_3_), 4.69 (dd, 1H, *J*_1_ = 6.9 Hz, *J*_2_ = 2.2 Hz, C3 β-lactam), 5.05 (d, 1H, *J* = 2.0 Hz, C4 β-lactam), 6.34 (d, 1H, *J* = 15.6 Hz, C*H*=CH–C(=O)–NH), 6.69 (d, 1H, *J* = 6.8 Hz, CH=CH–C(=O)–N*H*), 6.83 (d, 2H, *J* = 8.4 Hz Ar-*H*), 6.90 (t, 2H, Ar-*H*, *J*_1,2_ = 8.7 Hz), 7.03 (t, 2H, *J*_1,2_ = 8.6 Hz, Ar-*H*), 7.21–7.26 (m, 4H, Ar-*H*), 7.40–7.45 (m,2H, Ar-*H*), 7.58 (d, 1H, *J* = 15.6 Hz, CH=C*H*–C(=O)–NH); ^13^C NMR (75 MHz, CDCl_3_): −4.3 (Si–(*C*H_3_)_2_), 18.3 (*C*–(CH_3_)_3_), 25.7 (C–(*C*H_3_)_3_), 63.2 (C3 β-lactam), 66.4 (C4 β-lactam), 115.9 (d, *J* = 22.8 Hz, 4-F-*C*_6_H_4_), 116.0 (d, *J* = 21.8 Hz, 4-F-*C*_6_H_4_), 118.96 (*C*H=CH–C(=O)–NH), 119.13 (d, *J* = 8.0 Hz, 4-F-*C*_6_H_4_), 120.9 (4-OTBDMS-*C*_6_H_4_), 127.6 (4-OTBDMS-*C*_6_H_4_), 128.4 (4-OTBDMS-*C*_6_H_4_), 130.0 (d, *J* = 8.2 Hz, 4-F-*C*_6_H_4_), 130.8 (d, *J* = 3.3 Hz, 4-F-*C*_6_H_4_), 133.4 (d, *J* = 2.7 Hz, 4-F-*C*_6_H_4_), 141.4 (CH=*C*H–C(=O)–NH), 156.4 (4-OTBDMS-*C*_6_H_4_), 159.4 (d, *J* = 244.6 Hz, 4-F-*C*_6_H_4_), 163.8 (d, *J* = 250.7 Hz, 4-F-*C*_6_H_4_), 165.3 (*C*=O, β-lactam), 166.5 (*C*=O); HRMS for C_30_H_32_F_2_N_2_O_3_Si (*M*_r_ = 534.66899): calcd. *m*/*z* [M+H^+^] 535.2223, found 535.2225.

#### 4.2.3 1-[(3*R*,4*R*)-1-(4-Fluorophenyl)-4-(4-(*t*-butyldimethylsilyloxy)phenyl)-2-oxo-3-azetidinylamino]-2-phenyl-ethan-1-one(**4c**)

Obtained from **2** (44 mg, 0.11 mmol) and 2-phenylethanoyl chloride **3c** (35 mg, 0.22 mmol) as yellow oil (22 mg, yield 38%). [α]_D_^20^ +10 (*c* 10 mg/mL EtOAc); FT-IR (KBr) cm^−1^: 3307, 3063, 2956, 2930, 2858, 1761, 1654, 1608, 1510, 1387, 1265, 1228, 1141, 1011, 914, 834; ^1^H NMR (300 MHz, CDCl_3_): 0.18 (s, 6H, Si–(C*H*_3_)_2_), 0.96 (s, 9H, C–(C*H*_3_)_3_), 3.62 (s, 2H, C*H*_2_–C(=O)–NH), 4.55 (dd, 1H, *J*_1_ = 7.0 Hz, *J*_2_ = 2.1 Hz, C3 β-lactam), 4.85 (d, 1H, *J* = 2.0 Hz, C4 β-lactam), 6.50 (d, 1H, *J* = 6.8 Hz, CH_2_–C(=O)–N*H*), 6.79–6.88 (m, 4H, Ar-*H*), 7.13–7.17 (m, 4H, Ar-*H*), 7.25–7.35 (m, 5H, Ar-*H*); ^13^C NMR (75 MHz, CDCl_3_): −4.3 (Si–(*C*H_3_)_2_), 18.3 (*C*–(CH_3_)_3_), 25.7 (C–(*C*H_3_)_3_), 43.4 (*C*H_2_–C(=O)–NH), 63.3 (C3 β-lactam), 66.3 (C4 β-lactam), 115.9 (d, *J* = 22.5 Hz, 4-F-*C*_6_H_4_), 119.0 (d, *J* = 8.0 Hz, 4-F-*C*_6_H_4_), 120.8 (4-OTBDMS-*C*_6_H_4_), 127.6 (4-OTBDMS-*C*_6_H_4_), 127.7 (*C*_6_H_5_), 128.4 (4-OTBDMS-*C*_6_H_4_), 129.2 (*C*_6_H_5_), 129.6 (C_6_H_5_), 133.5 (d, *J* = 2.6 Hz, 4-F-*C*_6_H_4_), 134.2 (*C*_6_H_5_), 156.3 (4-OTBDMS-*C*_6_H_4_), 159.4 (d, *J* = 243.5 Hz, 4-F-*C*_6_H_4_), 164.1 (*C*=O, β-lactam), 171.6 (*C*=O); HRMS for C_29_H_33_FN_2_O_3_Si (*M*_r_ = 504.66782): calcd. *m*/*z* [M+H^+^] 505.2317, found 505.2309.

#### 4.2.4 1-[(3*R*,4*R*)-1-(4-Fluorophenyl)-4-(4-(*t*-butyldimethylsilyloxy)phenyl)-2-oxo-3-azetidinylamino]-2-(4-fluorophenyl)-ethan-1-one (**4d**)

Obtained from **2** (67 mg, 0.17 mmol) and 2-(4-fluorophenyl)ethanoyl chloride **3d** (60 mg, 0.34 mmol) as white solid (41 mg, yield 45%). mp 66–67 °C; [α]_D_^20^ +12 (*c* 10 mg/mL EtOAc); FT-IR (KBr) cm^−1^: 3310, 2956, 2931, 2858, 1760, 1654, 1608, 1510, 1387, 1265, 1227, 1157, 913, 834; ^1^H NMR (300 MHz, CDCl_3_): 0.18 (s, 6H, Si–(C*H*_3_)_2_), 0.96 (s, 9H, C–(C*H*_3_)_3_), 3.56 (s, 2H, C*H*_2_–C(=O)–NH), 4.60 (dd, 1H, *J*_1_ = 7.0 Hz, *J*_2_ = 2.0 Hz, C3 β-lactam), 4.83 (d, 1H, *J* = 2.0 Hz, C4 β-lactam), 6.73 (d, 1H, *J* = 7.0 Hz, CH_2_–C(=O)–N*H*), 6.79–6.87 (m, 4H, Ar-*H*), 7.01 (t, 2H, *J*_1,2_ = 8.6 Hz, Ar-*H*), 7.11–7.16 (m, 4H, Ar-*H*), 7.21–7.25 (m, 2H, Ar-*H*); ^13^C NMR (75 MHz, CDCl_3_): −4.3 (Si–(*C*H_3_)_2_), 18.3 (*C*–(CH_3_)_3_), 25.7 (C–(*C*H_3_)_3_), 42.4 (*C*H_2_–C(=O)–NH), 63.4 (C3 β-lactam), 66.2 (C4 β-lactam), 115.9 (d, *J* = 22.5 Hz, 4-F-*C*_6_H_4_), 116.0 (d, *J* = 21.4 Hz, 4-F-*C*_6_H_4_), 119.0 (d, *J* = 7.9 Hz, 4-F-*C*_6_H_4_), 120.8 (4-OTBDMS-*C*_6_H_4_), 127.5 (4-OTBDMS-*C*_6_H_4_), 128.3 (4-OTBDMS-*C*_6_H_4_), 130.0 (d, *J* = 3.3 Hz, 4-F-*C*_6_H_4_), 131.2 (d, *J* = 8.1 Hz, 4-F-*C*_6_H_4_), 133.4 (d, *J* = 2.8 Hz, 4-F-*C*_6_H_4_), 156.4 (4-OTBDMS-*C*_6_H_4_), 159.4 (d, *J* = 244.4 Hz, 4-F-*C*_6_H_4_), 162.3 (d, *J* = 246.1 Hz, 4-F-*C*_6_H_4_), 164.2 (*C*=O, β-lactam), 171.4 (*C*=O); HRMS for C_29_H_32_F_2_N_2_O_3_Si (*M*_r_ = 522.65829): calcd. *m*/*z* [M+H^+^] 523.2223, found 523.2211.

#### 4.2.5 [(3*R*,4*R*)-1-(4-Fluorophenyl)-4-(4-(*t*-butyldimethylsilyloxy)phenyl)-2-oxo-3-azetidinylamino]phenylformaldehyde (**4e**)

Obtained from **2** (46 mg, 0.12 mmol) and benzoyl chloride **3e** (33 mg, 0.24 mmol) as white solid (35 mg, yield 60%). mp 80–82 °C; [α]_D_^20^ +62 (*c* 10 mg/mL EtOAc); FT-IR (KBr) cm^−1^: 3339, 2956, 2930, 2857, 1762, 1653, 1510, 1388, 1264, 913, 833; ^1^H NMR (300 MHz, CDCl_3_): 0.20 (s, 6H, Si–(C*H*_3_)_2_), 0.97 (s, 9H, C–(C*H*_3_)_3_), 4.77 (dd, 1H, *J*_1_ = 6.4 Hz, *J*_2_ = 2.2 Hz, C3 β-lactam), 5.09 (d, 1H, *J* = 2.2 Hz, C4 β-lactam), 6.85 (d, 2H, *J* = 8.6 Hz, Ar-*H*), 6.90–6.96 (m, 3H, C(=O)–N*H*, Ar-*H*), 7.25–7.30 (m, 4H, Ar-*H*), 7.42–7.47 (m, 2H, Ar-*H*), 7.51–7.56 (m, 1H, Ar-*H*), 7.80–7.83 (m, 2H, Ar-*H*); ^13^C NMR (150 MHz, CDCl_3_): −4.2 (Si–(*C*H_3_)_2_), 18.3 (*C*–(CH_3_)_3_), 25.8 (C–(*C*H_3_)_3_), 63.6 (C3 β-lactam), 66.7 (C4 β-lactam), 116.0 (d, *J* = 22.8 Hz, 4-F-*C*_6_H_4_), 119.2 (d, *J* = 8.0 Hz, 4-F-*C*_6_H_4_), 120.9 (4-OTBDMS-*C*_6_H_4_), 127.4 (*C*_6_H_5_), 127.7 (4-OTBDMS-*C*_6_H_4_), 128.5 (4-OTBDMS-*C*_6_H_4_), 128.8 (*C*_6_H_5_), 132.4 (*C*_6_H_5_), 132.9 (*C*_6_H_5_), 133.6 (d, *J* = 1.8 Hz, 4-F-*C*_6_H_4_), 156.4 (4-OTBDMS-*C*_6_H_4_), 159.5 (d, *J* = 244.3 Hz, 4-F-*C*_6_H_4_), 164.4 (*C*=O, β-lactam), 167.5 (*C*=O); HRMS for C_28_H_31_FN_2_O_3_Si (*M*_r_ = 490.64124): calcd. *m*/*z* [M+H^+^] 491.2160, found 491.2161.

#### 4.2.6 [(3*R*,4*R*)-1-(4-Fluorophenyl)-4-(4-(*t*-butyldimethylsilyloxy)phenyl)-2-oxo-3-azetidinylamino](4-fluorophenyl)formaldehyde (**4f**)

Obtained from **2** (46 mg, 0.12 mmol) and 4-fluorbenzoyl chloride **3f** (38 mg, 0.24 mmol) as white solid (25 mg, yield 41%). mp 89–91 °C; [α]_D_^20^ +57 (*c* 10 mg/mL EtOAc); FT-IR (KBr) cm^−1^: 3352, 2956, 2931, 2858, 1761, 1653, 1606, 1510, 1472, 1265, 1232, 1157, 914, 834; ^1^H NMR (300 MHz, CDCl_3_): 0.20 (s, 6H, Si–(C*H*_3_)_2_), 0.97 (s, 9H, C–(C*H*_3_)_3_), 4.76 (dd, 1H, *J*_1_ = 6.4 Hz, *J*_2_ = 2.2 Hz, C3 β-lactam), 5.07 (d, 1H, *J* = 2.0 Hz, C4 β-lactam), 6.85 (d, 2H, *J* = 8.4 Hz, Ar-*H*), 6.90–6.96 (m, 3H, C(=O)–N*H*, Ar-*H*), 7.12 (t, 2H, *J*_1,2_ = 8.5 Hz, Ar-*H*), 7.23–7.29 (m, 4H, Ar-*H*), 7.80–7.85 (m, 2H, Ar-*H*); ^13^C NMR (75 MHz, CDCl_3_): −4.3 (Si–(*C*H_3_)_2_), 18.3 (*C*–(CH_3_)_3_), 25.8 (C–(*C*H_3_)_3_), 63.5 (C3 β-lactam), 66.6 (C4 β-lactam), 115.92 (d, *J* = 22.0 Hz, 4-F-*C*_6_H_4_), 115.99 (d, *J* = 22.8 Hz, 4-F-*C*_6_H_4_), 119.1 (d, *J* = 8.0 Hz, 4-F-*C*_6_H_4_), 120.9 (4-OTBDMS-*C*_6_H_4_), 127.7 (4-OTBDMS-*C*_6_H_4_), 128.3 (4-OTBDMS-*C*_6_H_4_), 129.0 (d, *J* = 3.2 Hz, 4-F-*C*_6_H_4_), 129.8 (d, *J* = 9.1 Hz, 4-F-*C*_6_H_4_), 133.4 (d, *J* = 2.8 Hz, 4-F-*C*_6_H_4_), 156.5 (4-OTBDMS-*C*_6_H_4_), 159.5 (d, *J* = 244.1 Hz, 4-F-*C*_6_H_4_), 164.5 (*C*=O, β-lactam), 165.3 (d, *J* = 253.5 Hz, 4-F-*C*_6_H_4_), 166.4 (*C*=O); HRMS for C_28_H_30_F_2_N_2_O_3_Si (*M*_r_ = 508.63171): calcd. *m*/*z* [M+H^+^] 509.2066, found 509.2060.

### 4.3 General procedure for the preparation of compounds **5a**–**f**

To a solution of **4a**–**f** (35 mM) in ethanol, 1 M HCl (5.5 equiv) was added and the mixture was stirred at room temperature overnight. After the completion of the reaction, distilled water (10 mL) was added to the reaction mixture and the resulting mixture was extracted with ethyl acetate (3 × 20 mL). Organic layers were collected, dried over Na_2_SO_4_ and the solvent was evaporated. The product was purified by silica gel column chromatography (hexane/ethyl acetate 1:1).

#### 4.3.1 (*E*)-*N*-((2*R*,3*R*)-1-(4-Fluorophenyl)-2-(4-hydroxyphenyl)-4-oxoazetidin-3-yl)cinnamamide (**5a**)

Obtained from **4a** (87 mg, 0.17 mmol) as white crystals (55 mg, yield 80%). mp 123–125 °C; [α]_D_^20^ +64 (*c* 5 mg/mL EtOAc); FT-IR (KBr) cm^−1^: 3283, 3059, 1740, 1656, 1615, 1509, 1388, 1348, 1224, 975, 832; ^1^H NMR (300 MHz, DMSO-*d*_6_): 4.72 (dd, 1H, *J*_1_ = 7.9 Hz, *J*_2_ = 2.4 Hz, C3 β-lactam), 5.07 (d, 1H, *J* = 2.4 Hz, C4 β-lactam), 6.63 (d, 1H, *J* = 15.8 Hz, C*H*=CH–C(=O)–NH), 6.76 (d, 2H, *J* = 8.3 Hz, Ar-*H*), 7.16 (t, 2H, *J*_1,2_ = 8.8 Hz, Ar-*H*), 7.23–7.29 (m, 4H, Ar-*H*), 7.39–7.50 (m, 4H, CH=C*H*–C(=O)–NH, Ar-*H*), 7.57–7.60 (m, 2H, Ar-*H*), 8.98 (d, 1H, *J* = 7.9 Hz, CH=CH–C(=O)–N*H*), 9.55 (s, 1H, Ar-O*H*); ^13^C NMR (150 MHz, DMSO-*d*_6_): 61.8 (C3 β-lactam), 65.6 (C4 β-lactam), 115.7 (4-OH-*C*_6_H_4_), 115.9 (d, *J* = 22.4 Hz, 4-F-*C*_6_H_4_), 118.7 (d, *J* = 7.9 Hz, 4-F-*C*_6_H_4_), 120.8 (*C*H=CH–C(=O)–NH), 126.6 (4-OH-*C*_6_H_4_), 127.7 (4-OH-*C*_6_H_4_), 128.0 (*C*_6_H_5_), 129.0 (*C*_6_H_5_), 129.8 (*C*_6_H_5_), 133.8 (4-F-*C*_6_H_4_), 134.5 (*C*_6_H_5_), 140.3 (CH=*C*H–C(=O)–NH), 157.7 (*C*=O), 158.2 (d, *J* = 240.7 Hz, 4-F-*C*_6_H_4_), 164.7 (4-OH-*C*_6_H_4_), 165.2 (*C*=O, β-lactam); HRMS for C_24_H_19_FN_2_O_3_ (*M*_r_ = 402.41766): calcd. *m*/*z* [M+H^+^] 403.1452, found 403.1457.

#### 4.3.2 (*E*)-3-(4-Fluorophenyl)-*N*-((2*R*,3*R*)-1-(4-fluorophenyl)-2-(4-hydroxyphenyl)-4-oxoazetidin-3-yl)acrylamide (**5b**)

Obtained from **4b** (74 mg, 0.14 mmol) as white crystals (53 mg, yield 92%). mp 117–119 °C; [α]_D_^20^ +61 (*c* 5 mg/mL EtOAc); FT-IR (KBr) cm^−1^: 3309, 1740, 1661, 1599, 1508, 1388, 1349, 1226, 1157, 976, 829; ^1^H NMR (300 MHz, DMSO-*d*_6_): 4.72 (dd, 1H, *J*_1_ = 8.1 Hz, *J*_2_ = 2.4 Hz, C3 β-lactam), 5.06 (d, 1H, *J* = 2.3 Hz, C4 β-lactam), 6.58 (d, 1H, *J* = 16.0 Hz, C*H*=CH–C(=O)–NH), 6.76 (d, 2H, *J* = 8.3 Hz, Ar-*H*), 7.16 (t, 2H, *J*_1,2_ = 8.8 Hz, Ar-*H*), 7.22–7.29 (m, 6H, Ar-*H*), 7.47 (d, 1H, *J* = 16.0 Hz, CH=C*H*–C(=O)–NH), 7.63–7.68 (m, 2H, Ar-*H*), 8.96 (d, 1H, *J* = 7.9 Hz, CH=CH–C(=O)–N*H*), 9.55 (s, 1H, Ar-O*H*); ^13^C NMR (75 MHz, DMSO-*d*_6_): 61.8 (C3 β-lactam), 65.6 (C4 β-lactam), 115.8 (4-OH-*C*_6_H_4_), 115.92 (d, *J* = 22.7 Hz, 4-F-*C*_6_H_4_), 115.97 (d, *J* = 21.7 Hz, 4-F-*C*_6_H_4_), 118.7 (d, *J* = 8.1 Hz, 4-F-*C*_6_H_4_), 120.7 (*C*H=CH–C(=O)–NH), 126.6 (4-OH-*C*_6_H_4_), 128.0 (4-OH-*C*_6_H_4_), 130.0 (d, *J* = 8.1 Hz, 4-F-*C*_6_H_4_), 131.2 (d, *J* = 3.1 Hz, 4-F-*C*_6_H_4_), 133.8 (d, *J* = 2.3 Hz, 4-F-*C*_6_H_4_), 139.2 (CH=*C*H–C(=O)–NH), 157.7 (*C*=O), 158.3 (d, *J* = 241.0 Hz, 4-F-*C*_6_H_4_), 162.9 (d, *J* = 248.1 Hz, 4-F-*C*_6_H_4_), 164.7 (4-OH-*C*_6_H_4_), 165.1 (*C*=O, β-lactam); HRMS for C_24_H_18_F_2_N_2_O_3_ (*M*_r_ = 420.40813): calcd. *m*/*z* [M+H^+^] 421.1358, found 421.1360.

#### 4.3.3 *N*-((2*R*,3*R*)-1-(4-Fluorophenyl)-2-(4-hydroxyphenyl)-4-oxoazetidin-3-yl)-2-phenylacetamide (**5c**)

Obtained from **4c** (40 mg, 0.08 mmol) as white crystals (27 mg, yield 88%). mp 185–187 °C; [α]_D_^20^ +15 (*c* 10 mg/mL EtOAc); FT-IR (KBr) cm^−1^: 3356, 3026, 1755, 1654, 1509, 1387, 1273, 1229, 1146, 832; ^1^H NMR (300 MHz, DMSO-*d*_6_): 3.50 (s, 2H, C*H*_2_–C(=O)–NH), 4.59 (dd, 1H, *J*_1_ = 7.6 Hz, *J*_2_ = 2.2 Hz, C3 β-lactam), 4.97 (d, 1H, *J* = 2.0 Hz, C4 β-lactam), 6.74 (d, 2H, *J* = 8.5 Hz, Ar-*H*), 7.13 (t, 2H, *J*_1,2_ = 8.7 Hz, Ar-*H*), 7.20–7.33 (m, 9H, Ar-*H*), 8.87 (d, 1H, CH_2_–C(=O)–N*H*, *J* = 7.9 Hz), 9.53 (s, 1H, Ar-O*H*); ^13^C NMR (75 MHz, DMSO-*d*_6_): 41.9 (*C*H_2_–C(=O)–NH), 61.6 (C3 β-lactam), 65.4 (C4 β-lactam), 115.9 (d, *J* = 22.8 Hz, 4-F-*C*_6_H_4_), 115.7 (4-OH-*C*_6_H_4_), 118.6 (d, *J* = 7.8 Hz, 4-F-*C*_6_H_4_), 126.5 (4-OH-*C*_6_H_4_), 126.6 (*C*_6_H_5_), 128.0 (4-OH-*C*_6_H_4_), 128.3 (*C*_6_H_5_), 129.1 (*C*_6_H_5_), 133.8 (d, *J* = 2.5 Hz, 4-F-*C*_6_H_4_), 135.6 (*C*_6_H_5_), 157.7 (*C*=O), 158.2 (d, *J* = 242.4 Hz, 4-F-*C*_6_H_4_), 164.7 (4-OH-*C*_6_H_4_), 170.6 (*C*=O, β-lactam); HRMS for C_23_H_19_FN_2_O_3_ (*M*_r_ = 390.40696): calcd. *m*/*z* [M+H^+^] 391.1452, found 391.1442.

#### 4.3.4 2-(4-Fluorophenyl)-*N*-((2*R*,3*R*)-1-(4-fluorophenyl)-2-(4-hydroxyphenyl)-4-oxoazetidin-3-yl)acetamide (**5d**)

Obtained from **4d** (77 mg, 0.15 mmol) as white crystals (51 mg, yield 84%). mp 103–105 °C; [α]_D_^20^ +14 (*c* 10 mg/mL EtOAc); FT-IR (KBr) cm^−1^: 3274, 1750, 1683, 1509, 1388, 1348, 1220, 1146, 984, 831; ^1^H NMR (300 MHz, DMSO-*d*_6_): 3.51 (s, 2H, C*H*_2_–C(=O)–NH), 4.58 (dd, 1H, *J*_1_ = 7.9 Hz, *J*_2_ = 2.5 Hz, C3 β-lactam), 4.97 (d, 1H, *J* = 2.4 Hz, C4 β-lactam), 6.74 (d, 2H, *J* = 8.6 Hz, Ar-*H*), 7.10–7.31 (m, 10H, Ar-*H*), 8.87 (d, 1H, *J* = 8.0 Hz, CH_2_–C(=O)–N*H*), 9.55 (s, 1H, Ar-O*H*); ^13^C NMR (75 MHz, DMSO-*d*_6_): 40.9 (*C*H_2_–C(=O)–NH), 61.6 (C3 β-lactam), 65.4 (C4 β-lactam), 115.0 (d, *J* = 20.8 Hz, 4-F-*C*_6_H_4_), 115.7 (4-OH-*C*_6_H_4_), 115.9 (d, *J* = 22.7 Hz, 4-F-*C*_6_H_4_), 118.7 (d, *J* = 8.2 Hz, 4-F-*C*_6_H_4_), 126.6 (4-OH-*C*_6_H_4_), 128.0 (4-OH-*C*_6_H_4_), 131.0 (d, *J* = 7.8 Hz, 4-F-*C*_6_H_4_), 131.7 (d, *J* = 3.4 Hz, 4-F-*C*_6_H_4_), 133.8 (d, *J* = 1.9 Hz, 4-F-*C*_6_H_4_), 157.7 (*C*=O), 158.2 (d, *J* = 240.5 Hz, 4-F-*C*_6_H_4_), 161.1 (d, *J* = 242.6 Hz, 4-F-*C*_6_H_4_), 164.7 (4-OH-*C*_6_H_4_), 170.6 (*C*=O, β-lactam); HRMS for C_23_H_18_F_2_N_2_O_3_ (*M*_r_ = 408.39743): calcd. *m*/*z* [M+H^+^] 409.1358, found 409.1357.

#### 4.3.5 *N*-((2*R*,3*R*)-1-(4-Fluorophenyl)-2-(4-hydroxyphenyl)-4-oxoazetidin-3-yl)benzamide (**5e**)

Obtained from **4e** (62 mg, 0.13 mmol) as white crystals (35 mg, yield 74%). mp 226–228 °C; [α]_D_^20^ +69 (*c* 10 mg/mL EtOAc); FT-IR (KBr) cm^−1^: 3294, 1741, 1635, 1508, 1396, 1225, 1170, 828; ^1^H NMR (300 MHz, DMSO-*d*_6_): 4.78 (dd, 1H, *J*_1_ = 8.0 Hz, *J*_2_ = 2.6 Hz, C3 β-lactam), 5.14 (d, 1H, *J* = 2.5 Hz, C4 β-lactam), 6.77 (d, 2H, *J* = 8.7 Hz, Ar-*H*), 7.17 (t, 2H, *J*_1,2_ = 8.9 Hz, Ar-*H*), 7.25–7.32 (m, 4H, Ar-*H*), 7.48–7.60 (m, 3H, Ar-*H*), 7.86–7.89 (m, 2H, Ar-*H*), 9.31 (d, 1H, *J* = 8.1 Hz, C(=O)–N*H*), 9.54 (s, 1H, Ar-O*H*); ^13^C NMR (75 MHz, DMSO-*d*_6_): 61.5 (C3 β-lactam), 65.8 (C4 β-lactam), 115.8 (4-OH-*C*_6_H_4_), 115.9 (d, *J* = 22.7 Hz, 4-F-*C*_6_H_4_), 118.6 (d, *J* = 8.2 Hz, 4-F-*C*_6_H_4_), 126.7 (4-OH-*C*_6_H_4_), 127.3 (4-OH-*C*_6_H_4_), 128.1 (*C*_6_H_5_), 128.6 (*C*_6_H_5_), 131.9 (*C*_6_H_5_), 133.0 (*C*_6_H_5_), 133.9 (d, *J* = 2.1 Hz, 4-F-*C*_6_H_4_), 157.7 (*C*=O), 158.2 (d, *J* = 240.4 Hz, 4-F-*C*_6_H_4_), 164.8 (4-OH-*C*_6_H_4_), 166.1 (*C*=O, β-lactam); HRMS for C_22_H_17_FN_2_O_3_ (*M*_r_ = 376.38038): calcd. *m*/*z* [M+H^+^] 377.1296, found 377.1291.

#### 4.3.6 4-Fluoro-*N*-((2*R*,3*R*)-1-(4-fluorophenyl)-2-(4-hydroxyphenyl)-4-oxoazetidin-3-yl)benzamide (**5f**)

Obtained from **4f** (81 mg, 0.16 mmol) as white crystals (45 mg, yield 71%). mp 235–237 °C; [α]_D_^20^ +63 (*c* 10 mg/mL EtOAc); FT-IR (KBr) cm^−1^: 3295, 1743, 1635, 1602, 1508, 1457, 1395, 1232, 1153, 828; ^1^H NMR (300 MHz, DMSO-*d*_6_): 4.77 (d, 1H, *J* = 7.9 Hz, C3 β-lactam), 5.12 (d, 1H, *J* = 2.4 Hz, C4 β-lactam), 6.77 (d, 2H, *J* = 7.7 Hz, Ar-*H*), 7.17 (t, 2H, *J*_1,2_ = 8.2 Hz, Ar-*H*), 7.24–7.37 (m, 6H, Ar-*H*), 7.92–7.97 (m, 2H, Ar-*H*), 9.33 (d, 1H, *J* = 7.7 Hz, C(=O)–N*H*), 9.54 (s, 1H, Ar-O*H*); ^13^C NMR (75 MHz, DMSO-*d*_6_): 61.5 (C3 β-lactam), 65.8 (C4 β-lactam), 115.6 (d, *J* = 21.7 Hz, 4-F-*C*_6_H_4_), 115.8 (4-OH-*C*_6_H_4_),116.0 (d, *J* = 22.6 Hz, 4-F-*C*_6_H_4_), 118.6 (d, *J* = 7.8 Hz, 4-F-*C*_6_H_4_), 126.7 (4-OH-*C*_6_H_4_), 128.1 (4-OH-*C*_6_H_4_), 129.5 (d, *J* = 2.8 Hz, 4-F-*C*_6_H_4_), 130.1 (d, *J* = 8.9 Hz, 4-F-*C*_6_H_4_), 133.9 (d, *J* = 2.5 Hz, 4-F-*C*_6_H_4_), 157.7 (*C*=O), 158.2 (d, *J* = 242.6 Hz, 4-F-*C*_6_H_4_), 164.3 (d, *J* = 251.8 Hz, 4-F-*C*_6_H_4_), 164.7 (4-OH-*C*_6_H_4_), 165.1 (*C*=O, β-lactam); HRMS for C_22_H_16_F_2_N_2_O_3_ (*M*_r_ = 394.37085): calcd. *m*/*z* [M+H^+^] 395.1202, found 395.1195.

### 4.4 Cell culture and stable transfection with human NPC1L1 protein

Madin-Darby Canine Kidney II wild type (MDCKIIwt), MDCKII cells stably expressing human NPC1L1 (hNPC1L1/MDCKII), and HepG2 cells were cultured in Dulbecco’s Modified Eagle Medium (DMEM) (Gibco®, Life Technologies, Carlsbad, USA) containing 10% fetal calf serum (FCS) (Sigma–Aldrich, Munich, Germany), 1% penicillin/streptomycin in a humidified 5% CO_2_ incubator at 37 °C. MDCKIIwt cells were stably transfected with human NPC1L1 using Lipofectamine LTX (Life Technologies, Carlsbad, USA) according to the manufacturer’s protocol. After 48 h, selection with 1000 μg/mL G418 (Sigma–Aldrich, Munich, Germany) was started. Selection lasted for two weeks, after which transfected cells were maintained in the medium containing 500 μg/mL G418.

### 4.5 Cytotoxicity determination

MDCKIIwt, hNPC1L1/MDCKII, and HepG2 cells were plated in 96-well microtiter plates at 2 × 10^4^ cells/mL on day 0. On the next day, tested compounds were added to obtain five 10-fold consecutive dilutions in range from 10^−8^ to 10^−4^ mol/L, and incubated for further 72 h. Cytotoxicity of the compounds was determined by the MTT cell proliferation assay (Sigma–Aldrich, Munich, Germany).^22^ The results are expressed as LC_50_ values. The absorbance (*A*) was measured on a microplate reader at 570 nm. Cytotoxicity of each compound was tested in quadruplicate in three independent experiments. Furthermore, toxicity of the compounds in combination with micelles was tested using the same method. MDCKIIwt and hNPC1L1/MDCKII were plated in 96-well microtiter plates (150 μL medium/well) at 2 × 10^5^ cells/mL 24 h before the experiment. Micelles were prepared according to the modified method described by Field et al.^23^ In brief, micellar components were mixed with DMEM supplemented with 10% FCS to obtain final concentrations of 0.25 mM oleic acid, 50 μM cholesterol, 10 μM compactin, 50 μM mevalonate, 5 mM Na-taurocholate (Sigma–Aldrich, Munich, Germany) and sonicated. Tested compounds (25, 50, 100, 150, 200 μM) were added to the prepared medium, vortexed and applied to the cells for 1 h. Cytotoxicity was determined using the MTT assay.

### 4.6 In vitro cholesterol uptake

In vitro cholesterol uptake was determined as described by Dražić et al.^19^ In brief, hNPC1L1/MDCKII cells were plated in 24-well plates at 5 × 10^5^ cells/mL medium (500 μL medium/well) in DMEM supplemented with 5% LPDS 24 h before the treatment. Cells were washed once with PBS and medium containing micelles, [^3^H]cholesterol (0.18 μCi/mL medium) ([1,2-3H(N)] cholesterol, 1 mCi/mL, ARC Inc., St. Louis, USA) and tested compounds in concentrations of 0–200 μM was added. After 1 h, the medium was removed, cells were washed three times with ice-cold 0.2% free fatty acid-free BSA and lysed. Radioactivity in the lysate (100 μL) was measured by liquid scintillation counting. Protein content in lysates was determined with the DC Protein Assay (Bio-Rad, Hercules, USA). The results were expressed as percentage of inhibition compared to untreated cells in CPM/mg protein. Activity of each compound was tested in duplicate in three independent experiments.

### 4.7 In vivo acute cholesterol absorption

26 weeks old male C57BL/6 mice (3–4 per group) were maintained in a temperature-controlled environment under a 12 h light/12 h dark cycle with free access to chow diet (Altromin Spezialfutter GmbH, Lage, Germany) and water. For two days, mice were treated with ezetimibe, tested compounds (20 mg/kg/day) or vehicle. For the treatment, compounds were dissolved in corn oil and administered to mice (100 μL) after 4 h fasting by oral gavage. On day 2, 90 min after compound administration, mice were gavaged with corn oil (200 μL) containing [^3^H]cholesterol (2 μCi) ([1,2-3H(N)] cholesterol, 1 mCi/mL, ARC Inc., St. Louis, USA). Liver and small intestines were collected 4 h post-gavage and cholesterol uptake and absorption were determined as previously described.^24^ Animal experiments were performed according to the standards set by the Austrian Federal Ministry of Science, Research, and Economy, Vienna, Austria.

### 4.8 Statistical analysis

The results are presented as mean ± SEM Statistical analysis was performed using one-way analysis of variance (ANOVA) followed by Dunnett’s test for multiple comparisons using (GraphPad Prism 5.0, San Diego, USA). Differences were considered significant at *p* <0.05.

## References

[1] Finegold J.A., Asaria P., Francis D.P. (2013). Int. J. Cardiol..

[2] Bruckert E. (2002). Cardiology.

[3] Ikonen E. (2006). Physiol. Rev..

[4] Lamon-Fava S. (2013). Curr. Opin. Lipidol..

[5] Burnett D.A. (1873). Curr. Med. Chem..

[6] Mikhailidis D.P., Lawson R.W., McCormick A.L., Sibbring G.C., Tershakovec A.M., Davies G.M., Tunceli K. (2011). Curr. Med. Res. Opin..

[7] Altmann S.W., Davis H.R., Zhu L.J., Yao X., Hoos L.M., Tetzloff G., Iyer S.P.N., Maguire M., Golovko A., Zeng M., Wang L., Murgolo N., Graziano M.P. (2004). Science.

[8] Wang Y., Zhang H., Huang W., Kong J., Zhou J., Zhang B. (2009). Eur. J. Med. Chem..

[9] Khan A., Safdar M., Khan M.M.A., Khattak K.N., Anderson R.A. (2003). Diabetes Care.

[10] Subash Babu P., Prabuseenivasan S., Ignacimuthu S. (2007). Phytomedicine.

[11] Lee J.-S., Jeon S.-M., Park E.-M., Huh T.-L., Kwon O.-S., Lee M.-K., Choi M.-S. (2003). J. Med. Food.

[12] Kannappan S., Jayaraman T., Rajasekar P., Ravichandran M.K., Anuradha C.V. (2006). Singapore Med. J..

[13] Viegas-Junior C., Danuello A., Da Silva Bolzani V., Barreiro E.J., Fraga C.A.M. (1829). Curr. Med. Chem..

[14] Poljak T., Molčanov K., Margetić D., Habuš I. (2008). Croat. Chem. Acta.

[15] Radolović K., Habuš I., Kralj B. (2009). Heterocycles.

[16] Kovač V., Radolović K., Habuš I., Sieber D., Heinze K., Rapić V. (2009). Eur. J. Inorg. Chem..

[17] Radolović K., Molčanov K., Habuš I. (2010). J. Mol. Struct..

[18] Vazdar K., Margetić D., Habuš I. (2011). Heterocycles.

[19] Dražić T., Molčanov K., Sachdev V., Malnar M., Hećimović S., Patankar J.V., Obrowsky S., Levak-Frank S., Habuš I., Kratky D. (2014). Eur. J. Med. Chem..

[20] Ojima I., Habuš I. (1990). Tetrahedron Lett..

[21] Weinglass A.B., Köhler M.G., Nketiah E.O., Liu J., Schmalhofer W., Thomas A., Williams B., Beers L., Smith L., Hafey M., Bleasby K., Leone J., Tang Y.S., Braun M., Ujjainwalla F., McCann M.E., Kaczorowski G.J., Garcia M.L. (2008). Mol. Pharmacol..

[22] Mosmann T. (1983). J. Immunol. Methods.

[23] Field F.J., Shreves T., Fujiwara D., Murthy S., Albright E., Mathur S.N. (1811). J. Lipid Res..

[24] Obrowsky S., Chandak P.G., Patankar J.V., Povoden S., Schlager S., Kershaw E.E., Bogner-Strauss J.G., Hoefler G., Levak-Frank S., Kratky D. (2013). J. Lipid Res..

