# Supplementary material for: Synthesis and evaluation of novel amide amino-β-lactam derivatives as cholesterol absorption inhibitors
Source: Bioorg Med Chem. 2015 May 15;23(10):2353–9. doi: 10.1016/j.bmc.2015.03.067 (PMC4414353; doi:10.1016/j.bmc.2015.03.067)
Supplement: Supplementary data — Supporting information. [file mmc1.docx]

**Supporting information**

**Synthesis and evaluation of novel amide amino-β-lactam derivatives as cholesterol absorption inhibitors**

Tonko Dražić,^a*^ Vinay Sachdev,^b^ Christina Leopold,^b^ Jay V. Patankar,^b,c^ Martina Malnar,^a^ Silva Hećimović,^a^ Sanja Levak-Frank,^b^ Ivan Habuš,^a^ Dagmar Kratky^b*^

^a^ Ruđer Bošković Institute, Bijenička cesta 54, Zagreb, Croatia

^b^ Institute of Molecular Biology and Biochemistry, Medical University of Graz, Graz, Austria

^c^ present address: Centre for Molecular Medicine and Therapeutics, Department of Medical

Genetics, University of British Columbia, Vancouver, Canada

Table of contents:

^1^H and ^13^C NMR spectra of compounds **4a**-**f** and **5a**-**f.**

Cytotoxicity of compounds **5a-f** in combination with micelles in MDCKIIwt and hNPC1L1/MDCKII cells: Figure S1


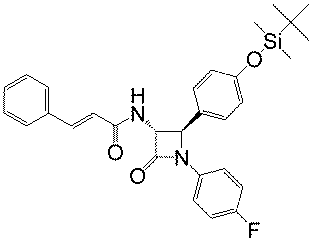


^1^H NMR spectrum of **4a**

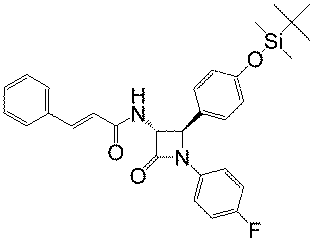


^13^C NMR spectrum of **4a**


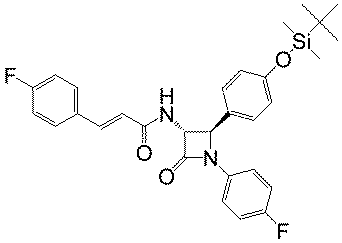


^1^H NMR spectrum of **4b**

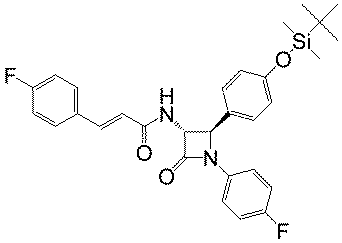


^13^C NMR spectrum of **4b**


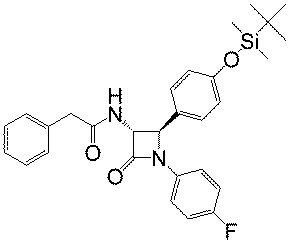


^1^H NMR spectrum of **4c**

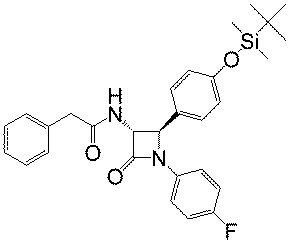


^13^C NMR spectrum of **4c**

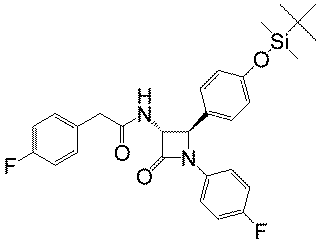


^1^H NMR spectrum of **4d**

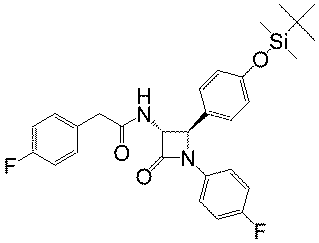


^13^C NMR spectrum of **4d**


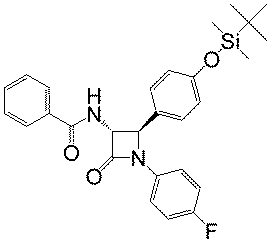


^1^H NMR spectrum of **4e**

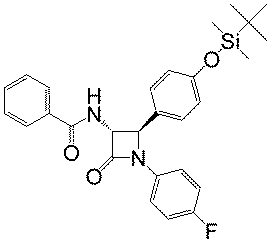


^13^C NMR spectrum of **4e**

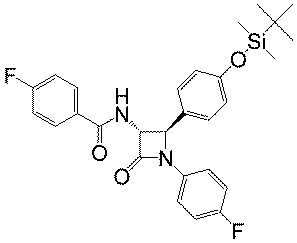


^1^H NMR spectrum of **4f**

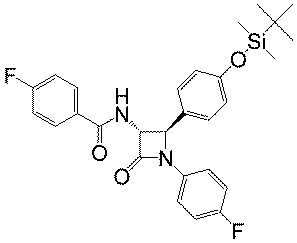


^13^C NMR spectrum of **4f**

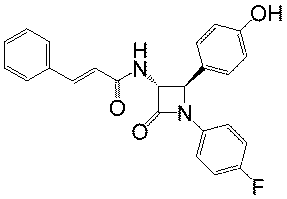


^1^H NMR spectrum of **5a**

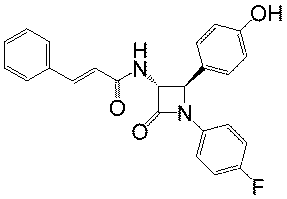


^13^C NMR spectrum of **5a**

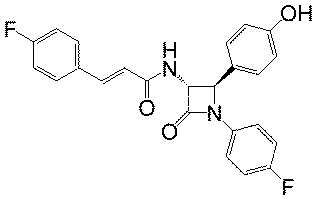


^1^H NMR spectrum of **5b**

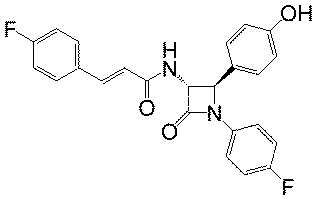


^13^C NMR spectrum of **5b**

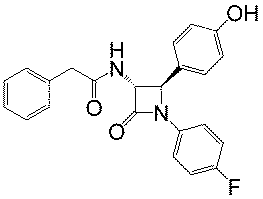


^1^H NMR spectrum of **5c**

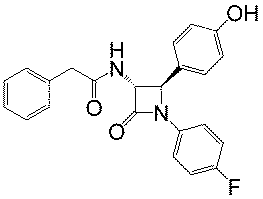


^13^C NMR spectrum of **5c**

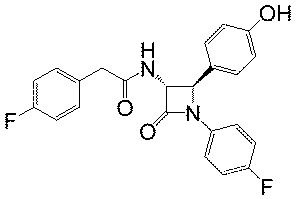


^1^H NMR spectrum of **5d**

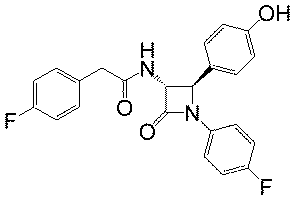


^13^C NMR spectrum of **5d**

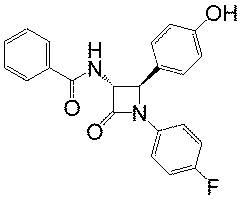


^1^H NMR spectrum of **5e**, contains remains of EtOAc and hexane

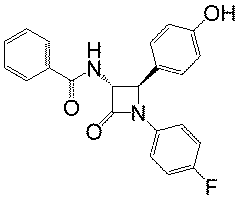


^13^C NMR spectrum of **5e**

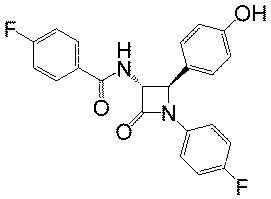


^1^H NMR spectrum of **5f**

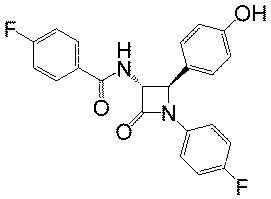


^13^C NMR spectrum of **5f**

**A B**


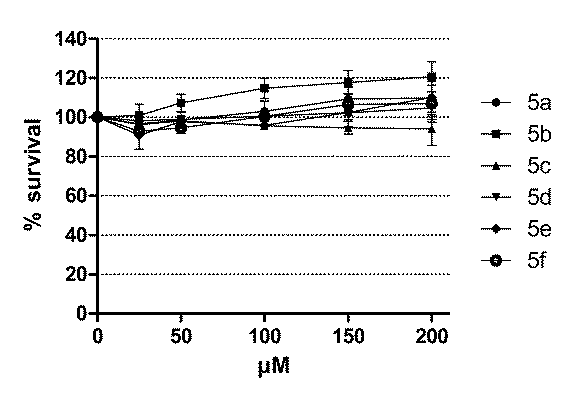

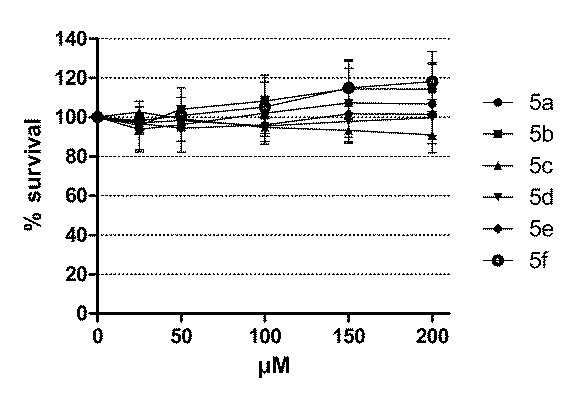


**Figure S1.** No cytotoxicity of compounds **5a-f** in combination with micelles in (**A**) MDCKIIwt and (**B**) hNPC1L1/MDCKII cells. The cells were incubated with the indicated concentrations of the compounds in Medium B (0.25 mM oleic acid, 50 μM free cholesterol, 10 μM compactin, 50 μM mevalonate, 5 mM Na-taurocholate in DMEM supplemented with 5% LPDS) for 1h. The results are expressed as percentage of the survival of cells treated with the compounds compared to untreated cells and represent mean ± S.E.M. of three independent experiments.
